# Development of nomogram and discussion of radiotherapy effect for osteosarcoma survival

**DOI:** 10.1038/s41598-023-27476-9

**Published:** 2023-01-05

**Authors:** Wu Xue, Ziyan Zhang, Haichi Yu, Chen Li, Yang Sun, Junyan An, Le Qi, Jun Zhang, Qinyi Liu

**Affiliations:** Department of Orthopedics, Second Affiliated Hospital of Jilin University, Changchun, People’s Republic of China

**Keywords:** Cancer, Oncology

## Abstract

This study aimed to develop a predictive system for prognostic evaluation of osteosarcoma patients. We obtained osteosarcoma sample data from 1998 to 2016 using SEER*Stat software version 8.3.8, and established a multivariable Cox regression model using R-4.0.3 software. Data were extracted from the Surveillance, Epidemiology, and End Results (SEER) database. The diagnosis of the model was completed through influential cases, proportionality, and multicollinearity. The predictive ability of the model was tested using area under the curve (AUC), calibration curves, and Brier scores. Finally, the bootstrap method was used to internally verify the model. In total, data from 3566 patients with osteosarcoma were included in this study. The multivariate Cox regression model was used to determine the independent prognostic variables. A nomogram and Kaplan–Meier survival curve were established. The AUC and Brier scores indicated that the model had a good predictive calibration. In addition, we found that the radiotherapy appears to be a risk factor of patients with osteosarcoma and made a discussion. We developed a prognostic evaluation system for patients with osteosarcoma for 1-, 3-, and 5-year overall survival with good predictive ability using sample data extracted from the SEER database. This has important clinical significance for the early identification and treatment of high-risk groups of osteosarcoma patients.

## Introduction

Osteosarcoma is the most common primary malignant bone tumor, accounting for approximately 35% of all primary malignant bone tumors^1^. They are composed of mesenchymal cells that produce bone-like tissues. Osteosarcomas can occur in any part of the bone. However, long bones such as pelvis, spine, and jaw are commonly affected^2^. Moreover, it is slightly more common in males than in females. The age of onset of osteosarcoma presents a bimodal distribution, mostly occurring in children and adolescents. One peak is noted at the age of 15–19 years, where the annual incidence is 8–11 cases/million persons^3^. The other peak is noted in the older individuals aged over 60 years^4^, where osteosarcoma occurs mostly secondary to Paget's disease, radiation bone disease, multiple hereditary osteochondromas, and bony fibrous dysplasia^2^.

For a long time, the treatment and prognosis of patients with osteosarcoma were unknown. Currently, we have found that the primary location, histological grade, treatment modality, and tumor diameter may affect the overall survival rate of patients with osteosarcoma. Before the 1970s, amputation was the main treatment for osteosarcoma; however, the postoperative survival rate was low^5^. Subsequently, with the development of neoadjuvant chemotherapy, radiotherapy, and surgical limb-salvage surgery, local progression of osteosarcoma has been effectively controlled and the postoperative relapse-free survival rate has increased to approximately 60%^6^.

Osteosarcoma progresses rapidly, with metastasis occurring in approximately 15–20% of patients at diagnosis^7,8^. Therefore, it is important to evaluate the prognosis of osteosarcoma at the time of diagnosis and perform early interventions. Furthermore, nomograms are widely used in cancer prognosis, and can reduce statistical predictive models into a single numerical estimate of the probability of an event. User-friendly graphical interfaces for generating these estimates facilitate the use of nomograms during clinical encounters to inform clinical decision^9^. This can provide a clinical reference for the early identification and targeted treatment of patients with high-risk osteosarcoma. The Surveillance, Epidemiology, and End Results (SEER) database is a publicly available cancer reporting system funded by the US federal government, representing population-based cancer information in 18 states^10^. The present study aimed to establish and verify a clinical predictive model, and develop a nomogram to predict the overall survival rate at 1, 3, and 5-year from sample data of eligible patients with osteosarcoma obtained through the SEER database.

## Methods

### Data source

SEER belong to public databases. The patients involved in the database have obtained ethical approval. Users can download relevant data for free for research and publish relevant articles. Our study is based on open source data, so there are no ethical issues and other conflicts of interest. We obtained sample data of osteosarcoma patients from 1998 to 2016 through SEERState 8.3.8. The data collected included sex, age at diagnosis, survival time, primary tumor site, treatment status, histological classification and grade, and the tumor diameter of the patients suffering from osteosarcoma.

### Inclusion and exclusion criteria

#### Inclusion criteria

Case samples diagnosed with osteosarcoma screened according to International Classification of Diseases for Oncology, Third Edition (ICD-O-3) histology/behavior: "osteosarcoma, not otherwise specified (NOS)" (9180/3), "chondroblastic osteosarcoma, NOS" (9181/3), "fibroblastic osteosarcoma" (9182/3), "telangiectatic osteosarcoma" (9183/3), "osteosarcoma in Paget’s disease of bone" (9184/3), "small cell osteosarcoma" (9185/3), "central osteosarcoma" (9186/3), "intraosseous well differentiated osteosarcoma" (9187/3), "parosteal osteosarcoma" (9192/3), "periosteal osteosarcoma" (9193/3), and "high grade surface osteosarcoma" (9194/3)" were included. Data was also selected by the adapted classification scheme for tumors of adolescents and young adults site recode/World Health Organization (WHO) 2008: 4.1 osteosarcoma.

#### Exclusion criteria

Case samples with unknown status of surgical treatment, radiotherapy, and chemotherapy; unknown primary tumor site; unknown histological classification and grade; and survival time.

### Interested variables

The demographic classification of the patients included age at diagnosis, gender, location of the primary tumor, tumor grade, treatment method, and tumor size. We groups the age at diagnosis as 0–14, 15–19, 20–39, 40–59, and ≥ 60 years, considering that the 15–19 and ≥ 60 years age groups demonstrate the peak incidence of osteosarcoma, combined with the clinical cut-off age of aggressive chemotherapy and completely chemotherapy courses at 40 and 60 years, respectively^11–13^. Primary tumor location was divided into upper limb (C40.0, long bones of upper limbs, scapula and associated joints), lower limb (C40.2, long bones of lower limbs and associated joints), and other sites (including bones of skull and face and associated joints and pelvic bones, sacrum, coccyx and associated joints) groups. The histological grade was divided into “low grade” (grade I and grade II), "Grade III" and "Grade IV", of which Grade III and Grade IV are collectively referred to as "high grade". Treatment methods included surgery or not, chemotherapy or not, and radiotherapy or not. Finally, tumor diameter size was divided into ≤ 8 cm, > 8 cm, and unknown size, using 8 cm as the limit, according to the criteria for tumor diameter in the tumor-node-metastasis classification of bone sarcoma^14^.

### Statistical analysis

In this study, we used a multivariate Cox proportional hazard regression model. Furthermore, we used two-way stepwise method to screen variables to simplify the model. Subsequently, we fitted the model to obtain a final clinical predictive osteosarcoma prognosis model. We calculated the linear predictor (lp) and predictive probability. The model was completed through influential cases, proportionality, and multicollinearity. We used the receiver operator characteristic (ROC) to calculate AUC, where AUC > 0.7 indicated that the model has good discrimination. Furthermore, we used the calibration curves for 1, 3, and 5 years to obtain Brier scores (BSs), where BS < 0.5 indicated better predictive calibration. We developed 1-, 3-, and 5-year nomogram and survival curves, and then performed Kaplan–Meier (K–M) survival analysis for each impact variable. We used the bootstrap method to internally verify the established prediction model and performed sampling with replacement to establish a dataset with the same sample size in the model development cohort as the training set. The above-mentioned modeling process was performed on the training set, and its model performance was tested in the original model development cohort (AUCs and BSs were calculated). The above process was repeated 100 times to obtain 100 model performances and calculate the average value of AUC and BS as the internal verification model performance. *p* < 0.05 was considered to be statistically significant. We were performed all methods using the R-4.0.3 software.

### Ethics approval

SEER belong to public databases. The patients involved in the database have obtained ethical approval. Users can download relevant data for free for research and publish relevant articles. Our study is based on open source data, so there are no ethical issues and other conflicts of interest.

## Results

### Demographic baseline characteristics

A total of 3566 osteosarcoma cases, diagnosed between 1998 and 2016, were included in our study according to the inclusion and exclusion criteria. Of them, 1932 patients (54.18%) were male and 1634 patients (45.82%) were female. The average follow-up time was 62.5 months. In all cases, a high proportion of high-grade malignant tumors were noted: 1060 cases (29.73%) were grade III and 2060 cases (57.77%) were of grade IV. Regarding the treatment modality, a total of 3159 (88.33%) patients underwent surgery, 2770 (77.68%) patients received chemotherapy, and 384 (10.77%) patients received radiotherapy. Among them, 253(7.09%) patients received both chemotherapy and radiotherapy. Due to the particularity of radiotherapy results in this study, we focus on the relevant demographic characteristics of the 384 patients receiving radiotherapy in Table 1.

**Table 1:** Characteristics of patients undergone radiotherapy.

Variables	N	*p* value^a^
**Age**^b^		*p* < 0.05
0–39	134	
≥ 40	250	
**Sex**		
Male	204	
Female	180	
**Primary site**		*p* < 0.05
Upper limb	35	
Lower limb	71	
Other sites	278	
**Surgery**		*p* < 0.05
None	102	
Yes	282	
**Histologic type**		*p* < 0.05
Osteosarcoma	267	
Chondroblastic	70	
Other type	47	
**Grade**		*p* < 0.05
Low grade (G1 + G2)	30	
G3	135	
G4	219	
**Chemotherapy**		*p* < 0.05
None	131	
Yes	253	
**Tumor size**		*p* < 0.05
≤ 8 cm	167	
> 8 cm	136	
Unknown	81	

^a^*p* < 0.05 mains significantly different.

^b^According to the clinical cut-off age of aggressive chemotherapy at 40, we divide age groups into 0–39 and ≥ 40.

### Multivariate Cox's regression results and K–M survival analysis

**Figure 1:**
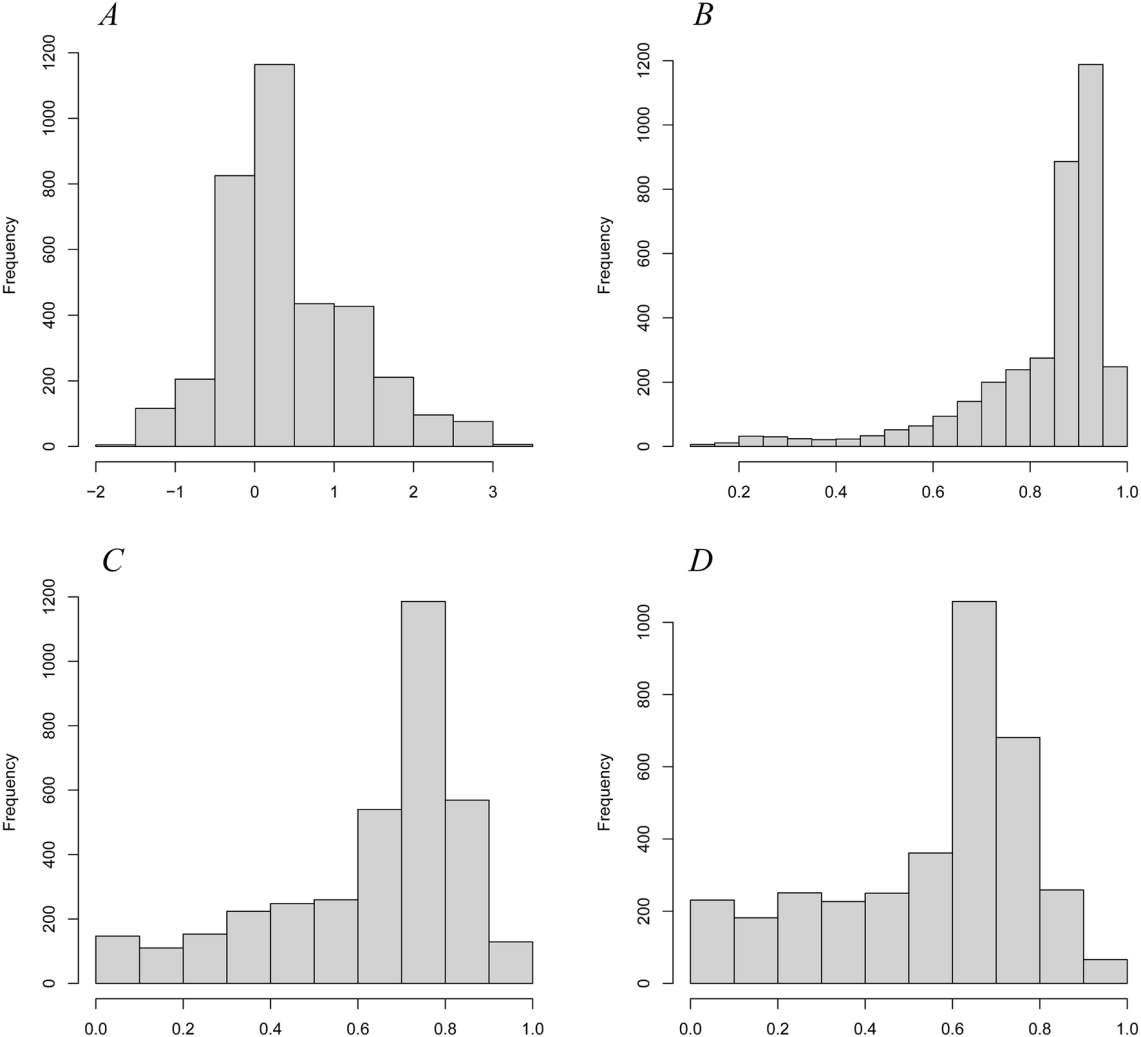
Linear predictor (**A**) and predicted probability (**B**–**D**). (**B**–**D)** Shows 1-year, 3-year, and 5-year predicted probability, respectively.

**Figure 2:**
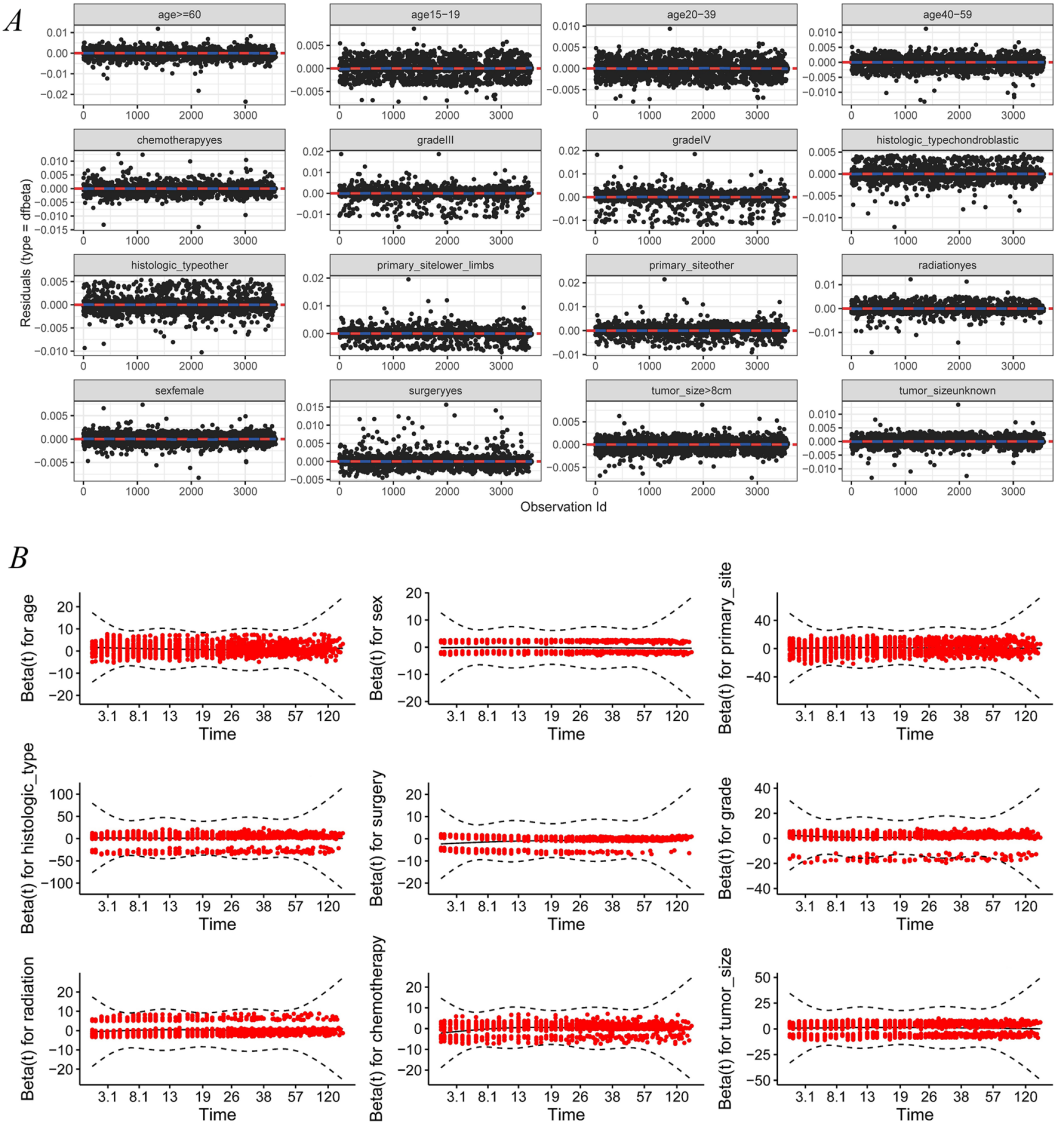
The influential case (**A**) and proportionality (**B**).

**Figure 3:**
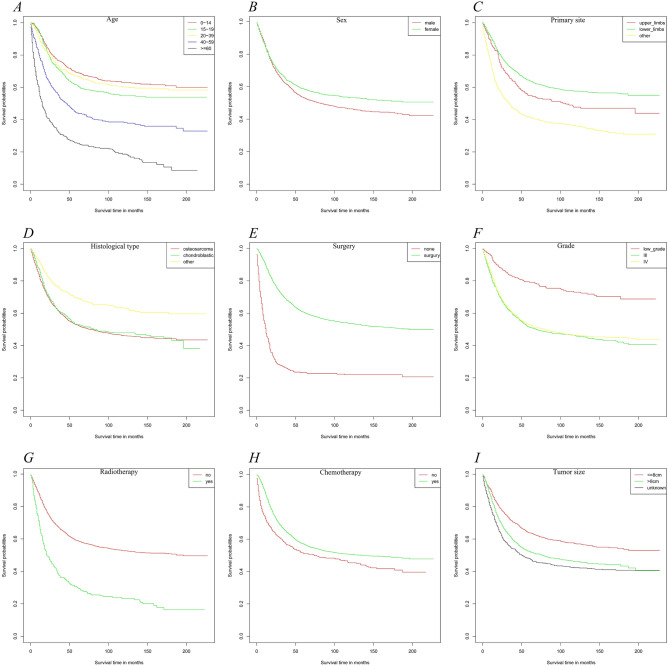
Kaplan–Meier estimated specific survival curve in patients with osteosarcoma stratified by age (**A**), sex (**B**), primary site (**C**), histological type (**D**), surgery (**E**), grade (**F**), radiation (**G**), chemetherapy (**H**), tumor size (**I**).

The parameters, including age at diagnosis, primary tumor site, tumor histological classification and grade, treatment (surgery, chemotherapy, and radiotherapy), and tumor size, were included the multivariate Cox regression analysis. We used the two-way stepwise method to fit the model and determine the independent prognostic variables. The demographic baseline and clinicopathological characteristics of all cases and the final multivariate Cox regression analysis results are presented in Table 2. We calculated the linear predictor of the Cox regression model and then calculated the predictive probability using the predicted value (Fig. 1). In addition, we completed the model diagnosis by testing the influential case, proportionality (Fig. 2), and multicollinearity. The influential case (Fig. 2a) of all cases in the model was within the acceptable range (|dfbeta| < 2δ), and proportionality (Fig. 2b) was also valid. For the multicollinearity test, the variance inflation factor (vif) of all variables was lower than 10, confirming that there was no multicollinearity in the model parameters. The following are the variables: age (1.3933), sex (1.0167), primary site (1.2763), histological type (1.0474), grade (1.1286), surgery (1.0983), radiation (1.1398), chemotherapy (1.2993), and tumor size (1.0976). Specific survival curves of patients with osteosarcoma were generated using the K–M method (Fig. 3).

**Table 2:** Patient characteristics and Selected variables in the SEER^a^ by multivariate Cox’s regression analysis.

Variables	N	Multivariate analysis		
		HR^b^	95% CI	*p* value^c^
**Age**				
0–14	910	Reference		
15–19	677	1.203	1.013–1.428	*p* < 0.05
20–39	799	1.202	1.010–1.431	*p* < 0.05
40–59	608	2.131	1.794–2.532	*p* < 0.05
≥ 60	572	3.509	2.926–4.210	*p* < 0.05
**Sex**				
Male	1932	Reference		
Female	1634	0.889	0.802–0.985	*p* < 0.05
**Primary site**				
Upper limb	392	Reference		
Lower limb	2006	0.809	0.682–0.959	*p* < 0.05
Other sites	1168	1.113	0.928–1.334	*p* < 0.05
**Surgery**				
None	461	Reference		
Yes	3105	0.364	0.320–0.414	*p* < 0.05
**Histologic type**				
Osteosarcoma	2377	Reference		
Chondroblastic	531	1.058	0.917–1.219	
Other type	658	0.799	0.686–0.931	*p* < 0.05
**Grade**				
Low grade (G1 + G2)	446	Reference		
G3	1060	2.584	2.058–3.244	*p* < 0.05
G4	2060	2.683	2.153–3.342	*p* < 0.05
**Radiation**				
None	384	Reference		
Yes	3182	1.311	1.135–1.515	*p* < 0.05
**Chemotherapy**				
None	796	Reference		
Yes	2770	0.886	0.775–1.014	0.07
**Tumor size**				
≤ 8 cm	1303	Reference		
> 8 cm	1434	1.551	1.375–1.515	*p* < 0.05
Unknown	829	1.413	1.227–1.627	*p* < 0.05

^a^Surveillance, Epidemiology, and End Results.

^b^Hazard ratio.

^c^*p* < 0.05 mains significantly different.

### Performance and validation of the model

**Figure 4:**
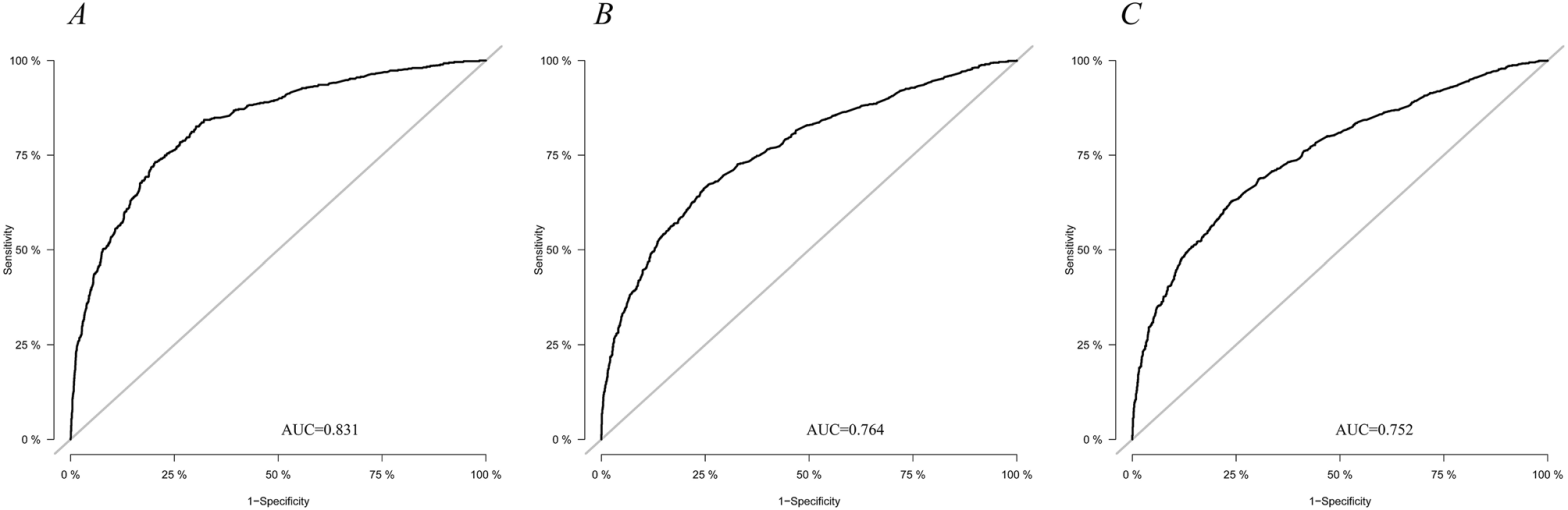
ROC curves. ROC curve analyses were generated to test the performance evaluating of the established predictive model, by the areas under the ROC curves (AUC). (**A**–**C**) ROC of 1-, 3- and 5- year, respectively.

**Figure 5:**
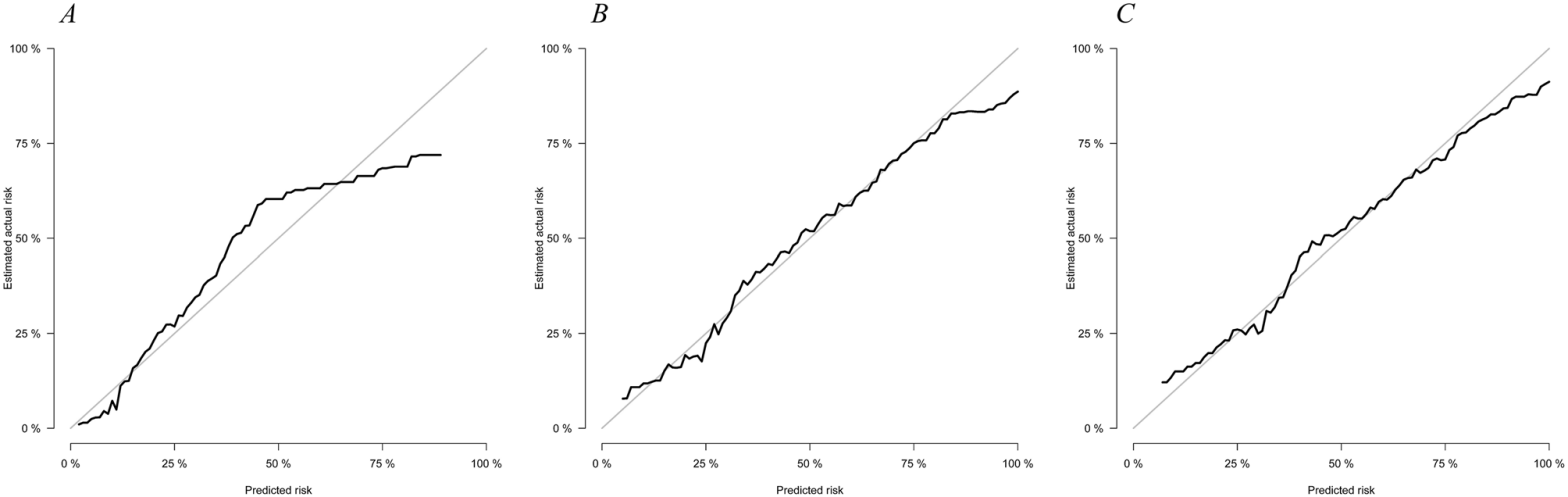
Calibration curves for 1- (**A**), 3- (**B**) and 5-year (**C**) survival. Calibration curves depict the calibration of each model in terms of the agreement between the predicted probabilities and observed outcomes of the training set.

Based on the model we developed, we plotted the ROC (Fig. 4) and calibration curve (Fig. 5) for 1, 3, and 5 years. The AUCs obtained were 0.831, 0.764, and 0.752, and the BSs were 0.104, 0.181, and 0.197, respectively. In this study, we adopted a bootstrap resampling method to verify the model. In internal verification, the calculated AUC were 0.828, 0.756, and 0.745, respectively, indicating that our model had good discrimination. Furthermore, the BS values were 0.105, 0.184, and 0.199, respectively, indicating that the model had good predictive calibration.

### Nomogram construction

**Figure 6:**
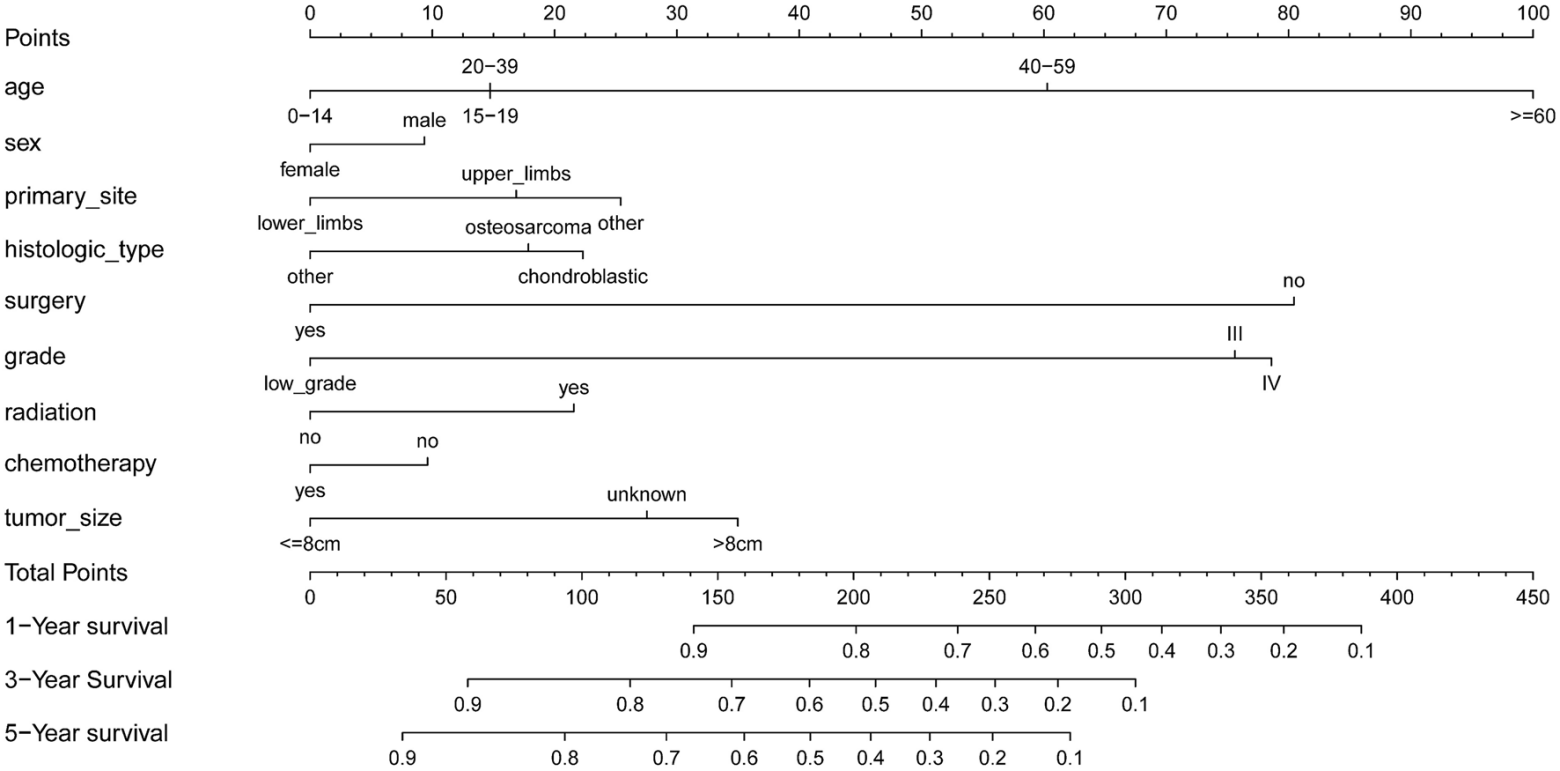
Nomogram predicting 1-, 3-, 5-year.

We established a nomogram for the 1-year, 3-year, and 5-year overall survival rates using the final result of multivariate Cox regression analysis, using R software (Fig. 6). Each variable determined the corresponding score on the top scale. The scores ranged from 0 to 100 points. All scores were summed to yield the total points of variables, projected vertically downward, to obtain the 1-, 3-, and 5-year survival rates. According to the nomogram, we found that age at diagnosis was the most important variable affecting survival rate, followed by a non-surgical approach, histologically high-malignant grade, tumor diameter > 8 cm, other primary sites, chondroblastic osteosarcoma, no chemotherapy, and male sex. Osteosarcoma and chondroblastic differences in histological classification were not large. Radiotherapy emerged as a risk factor for the prognosis of osteosarcoma.

## Discussion

Osteosarcoma is a rapidly progressive systemic disease. In the early stage of onset, intermittent local pain is often ignored. However, as the disease progresses rapidly, it results in persistent severe pain accompanied by fatigue, weight loss, low-grade fever, and systemic symptoms such as anemia. Approximately 15–20% of the patients have signs of metastasis at the time of diagnosis, of which 75% have lung metastases^7,8^. In recent decades, with the emergence of new neoadjuvant chemotherapy and radiotherapy techniques combined with limb-salvage surgery, the treatment and survival rates of osteosarcoma have greatly improved. In our study, we established a nomogram based on the osteosarcoma data in the SEER database, which visualized the abstract data, through Cox regression analysis, and derived a relationship between the parameters of osteosarcoma and its prognosis. This nomogram enables the calculation of the year-by-year survival rate by incorporating the relevant data, which can be used to distinguish the high-risk population during early stages of the diagnosis. Clinicians can perform targeted treatment and effective postoperative management according to the patient’s risk factors.

Osteosarcoma is the most common primary malignant tumor of the bone and accounts for approximately 35% of all primary bone malignant tumors. In adolescence, the incidence of osteosarcoma gradually increases^15^. The sudden increase in the incidence of osteosarcoma during puberty and faster-growing sites and its prevalence in males (60%) indicates that the occurrence of osteosarcoma may be related to the rapid growth of bones^3,7^. Although the incidence of osteosarcoma is highest in children and adolescents, its long-term survival rate can significantly increase after effective treatment. However, the survival rate of older patients is much lower than that of young patients^11,16–18^. Our findings that advanced age is the most important risk factor for the survival rate of patients with osteosarcoma also support this view. This is mainly because of the poor general condition of older individuals and the presence underlying diseases. Once osteosarcoma occurs, prognosis is relatively poor. In the present study, 88.47% of the patients over 60 years of age had high-grade tumors. Due to tumor metastasis, predilection for axial localization, and body intolerance, complete resection is almost impossible^19^. However, owing to factors such as reduced bone marrow tolerance, renal function, or insufficiency of cardiac function in older patients, chemotherapy is not commonly used^19,20^. According to our research, the prognosis of older patients can be judged at an early stage of diagnosis to guide treatment plans and improve the long-term survival rate of patients.

In the present study, the survival rate of patients who underwent radiotherapy was significantly lower than those who did not (*p* < 0.05), which confused us most. Although similar results have been reported in other established osteosarcoma models^21,22^, they have not been discussed in detail. As we all known, osteosarcoma has been thought to be radioresistant for a long time, which is not sensitive to radiotherapy. Currently, radiotherapy is not used as the first-line treatment of it. As a traditional treatment for osteosarcoma, complete surgical resection with clear margins is the most critical treatment for local osteosarcoma^7,23^. However, as a systemic cancer, a great deal of patients suffer distant metastasis of osteosarcoma^24^. In the early stages of cancer, micro-metastases, which cannot be resected completely by surgery, may develop in the lungs. Circulating tumor cells and micrometastases that are already present at the time of diagnosis require high-dose systemic therapy^25^. Therefore, pre- and post-operative chemotherapy combined with in situ resection is usually used for the treatment of osteosarcoma patients^3,25^. However, clinically, aggressive chemotherapy (i.e., regimens containing doxorubicin/cisplatin and high-dose methotrexate) is usually not used in patients older than 40 years^12,13^, which significantly reduces the efficacy of chemotherapy in this patient population. Considering the above reasons, physicians also use radiotherapy to make up for the lack of efficacy of surgery and chemotherapy during the treatment of special osteosarcoma patients. According to the research of scholars, the combination of neoadjuvant chemotherapy with radiotherapy obtained satisfactory osteosarcoma control effects. Machak et al.^26^ examined 187 patients with non-metastatic limb osteosarcoma who received neoadjuvant chemotherapy and local radiotherapy. Among them, 156 underwent surgery to remove the tumor tissue. After a 5-year follow-up period, the overall survival rate was 61%. It is worth noting that the 5-year survival rate of patients who had a pronounced response during treatment reached 90%. This result suggested that for non-metastatic osteosarcoma, surgical resection combined with adjuvant chemotherapy and local radiotherapy achieved satisfactory long-term survival rates. However, due to the invasion and rapid metastasis of osteosarcoma, as well as the complexity of the primary site and patients' condition, not all patients can undergo complete resection of the tumor. In fact, there is a significant correlation between incomplete surgical resection and poor prognosis^27–29^. Fortunately, the application of radiotherapy has greatly improved the local control rate in patients who cannot undergo surgery or have inadequate margins for various reasons, and poorly treatment effect of chemotherapy as well^30^. Ueda et al.^31^ analyzed data from 275 patients with primary osteosarcoma. In their study, the local control rate was 68% within 5 years in patients with unresectable or positive margins who received radiotherapy. Preoperative radiotherapy can reduce tumor volume, facilitate complete resection of local tumors, and provide conditions for limb-salvage surgery^32^. Meanwhile, chemotherapy is combined with preoperative radiotherapy to enhance the local effect of radiotherapy. However, for patients with serious underlying diseases or the elderly, chemotherapy alone cannot achieve the desired therapeutic effects^32,33^. On the contrary, it can produce toxic effects, and it is this recommended to use radiotherapy and chemotherapy simultaneously or use radiotherapy after chemotherapy, which can have systematic anticancer and local radiosensitizing effects^23,34^. In short, although radiotherapy is currently a non-first-line treatment for osteosarcoma, it can still be used as a palliative treatment to prolong the long-term survival rate for some of the above-mentioned special cases, such as incomplete surgical resection and poor chemotherapy effect^32^.

As we all known, radiotherapy have a long-term carcinogenic side effect^35,36^. Based on the most commonly affected sites of osteosarcoma, local radiotherapy for patients with osteosarcoma may increase the risk of secondary extraskeletal osteosarcoma and brain tumors^37,38^. Maruyama et al.^39^ believe that radiotherapy is very important for the treatment of osteosarcoma, but the radiation of the primary tumor may cause fibrosis and sclerosis of the radiation field and also lead to some secondary tumors with a local recurrence rate of up to 45%. Although there are some case reports that about thousandth patients occurred sarcoma after radiation exposure^40–42^, there is no direct evidence or mechanistic study that radiation therapy reduces long-term survival in patients with osteosarcoma. More importantly, in the present study, of the 384 patients who received both radiotherapy and chemotherapy, 250 (65.1%) patients were older than 40 years. As mentioned earlier, these patients are not able to receive high-dose chemotherapy, or even a full course of chemotherapy. At the same time, with the increase of age, patients' immune function also gradually declines. Meanwhile, 91.96% of received radiotherapy patients in our study were histologically high-level grade, which was reported has a high relapse rate with a long-term survival rate of only 20–30%^4,7,43^. They all received surgery, and 65.38% of cases received chemotherapy. The above situation indicated that the efficacy of surgery and chemotherapy is unsatisfied for patients aged over 40 years and with high-grade osteosarcoma. This just showed that radiation might be a palliative treatment method of choice to control tumor invasion and metastasis in the case of poor surgical resection and chemotherapy effects, to prolong the survival time of critical patients as much as possible. Among the patients receiving radiotherapy, middle-aged and elderly patients and high-grade osteosarcoma patients accounted for a high proportion, resulting in poor chemotherapy efficacy, increased radiation dose, and high tumor recurrence rate. These factors constituted confounding factors for the variable "radiotherapy", which led us to the conclusion that patients who received radiotherapy had poor survival rate. Combined with the research of other scholars mentioned above, we believe that although radiotherapy is generally not first-line treatment, it is still effective for local control of osteosarcoma in some special cases. For patients with poor effect of chemotherapy or surgical resection and those who are unable to undergo surgical treatment, we suggest the use of radiotherapy to locally control the tumor, prolong and improve the survival time, and even achieve long-term tumor survival. For high-risk patients, radiotherapy can also be performed before and during surgery to reduce the size of the primary tumor and inhibit tumor metastasis, thus providing conditions for negative surgical margins. Therefore, although the results of multivariate Cox regression analysis showed that the survival rate of patients who received radiotherapy was significantly lower than that of patients who did not, there was no direct evidence that radiotherapy was an independent risk factor for patients with osteosarcoma. On the contrary, radiotherapy has obvious efficacy in the palliative treatment of patients.

This study used the SEER database to obtain relevant osteosarcoma cases. Multivariate Cox regression was performed, a visual nomogram was plotted, and internal verification and K–M survival analysis were performed. The final AUC, calibration curves, and BS showed that the predictive model had a good ability to predict the prognosis of osteosarcoma at 1, 3, and 5 years. In this study, different from others, we used the two-way stepwise regression method to develop the model. This eliminated variables with insignificant effects, established the optimal regression equation, and obtained significant independent influencing factors. To confirm the accuracy of the model, a model that contains influential cases, proportionality, and multicollinearity was tested. In the internal verification of the model, we did not use the random split method. Instead, we chose the bootstrap method to establish 100 sets of models through repeated sampling with replacement to obtain 100 model performances and obtain the final internal verification by calculating the average value. Most importantly, we performed a systematic and comprehensive analysis of abnormal radiotherapy outcomes in our model, addressed possible reasons for poor survival in patients who underwent radiotherapy, and concluded that radiotherapy could not be defined as an independent risk factor. Although similar results have been published in other established osteosarcoma models, a detailed analysis was not performed.

Nevertheless, our research has some limitations. First, the present study was based on the SEER database that included data of American patients, resulting in a large proportion of Whites and Blacks and a small proportion of Asians, making our model's predictions for Asian populations biased. Additionally, in our study, an internal verification method was adopted, which lacks external verification to confirm the reliability of the model. Furthermore, our study included data on osteosarcoma samples from 1998 to 2016. During this period, the differences in diagnostic imaging and treatment methods also differed, which may have biased the prediction of results. Thirdly, we screened the samples in the database and excluded cases that did not meet the inclusion conditions and had missing data, which can produce a certain selection bias. Finally, from 1998 to 2016, radiotherapy technology continued to develop, from Intensity Modulated Radiation Therapy(IMRT) to Image Guided Radiation Therapy(IGRT), and then proton therapy was widely used in clinical practice^44,45^. Unfortunately, we did not include the patient's details of radiotherapy when collecting data, which could be a potential bias in considering radiotherapy as an independent risk factor.

## Conclusion

We used the sample cases obtained from the SEER database to establish and verify the 1-, 3-, and 5-year prognostic models of osteosarcoma patients through Cox regression analysis, establish a visual nomogram, and verify the predictive ability of the model. Although our study has certain limitations, it can provide clinically accurate personalized prediction results for the survival rate of patients with osteosarcoma. Although the results showed that the survival rate of patients who received radiotherapy was lower than that of patients who did not, we believe that radiotherapy cannot be defined as an independent risk factor for patients with osteosarcoma. In our model, the variable "radiotherapy" was influenced by confounding factors to draw conclusions that are quite different from reality. Other studies also suggested that radiotherapy plays an important role in special cases of osteosarcoma, such as when the tumor cannot be completely resection for technical or medical reasons, or when chemotherapy cannot obtain satisfactory control effects. Therefore, radiotherapy can be a palliative treatment to enhance chemotherapy sensitivity, improve the quality of life and even prolong the survival time of patients. For some special patients, systemic chemotherapy combined with local radiotherapy can avoid the use of high-dose chemotherapy drugs and reduce the short-term toxicity.

## Data Availability

Publicly available datasets were analyzed in this study. This data can be found here: Surveillance, Epidemiology, and End Results (SEER) database (https://seer.cancer.gov/).
